# The acceleration of cardiomyogenesis in embryonic stem cells *in vitro* by serum depletion does not increase the number of developed cardiomyocytes

**DOI:** 10.1371/journal.pone.0173140

**Published:** 2017-03-13

**Authors:** Katarzyna Anna Radaszkiewicz, Dominika Sýkorová, Lucia Binó, Jana Kudová, Markéta Bébarová, Jiřina Procházková, Hana Kotasová, Lukáš Kubala, Jiří Pacherník

**Affiliations:** 1 Department of Experimental Biology, Faculty of Science, Masaryk University, Brno, Czech Republic; 2 Department of Free Radical Pathophysiology, Institute of Biophysics, Academy of Sciences of the Czech Republic, Brno, Czech Republic; 3 International Clinical Research Center–Centre of Biomolecular and Cellular Engineering, St. Anne's University Hospital, Brno, Czech Republic; 4 Department of Physiology, Faculty of Medicine, Masaryk University, Brno, Czech Republic; 5 Department of Histology and Embryology, Faculty of Medicine, Masaryk University, Brno, Czech Republic; University of Tampere, FINLAND

## Abstract

The differentiation of pluripotent embryonic stem (ES) cells into various lineages *in vitro* represents an important tool for studying the mechanisms underlying mammalian embryogenesis. It is a key technique in studies evaluating the molecular mechanisms of cardiomyogenesis and heart development and also in embryotoxicology. Herein, modest modifications of the basic protocol for ES cell differentiation into cardiomyocytes were evaluated in order to increase the yield and differentiation status of developed cardiomyocytes. Primarily, the data show that ES cell cultivation in the form of non-adherent embryoid bodies (EBs) for 5 days compared to 8 days significantly improved cardiomyogenic differentiation. This is illustrated by the appearance of beating foci in the adherent EBs layer at earlier phases of differentiation from day 10 up to day 16 and by the significantly higher expression of genes characteristic of cardiomyogenic differentiation (sarcomeric alpha actinin, myosin heavy chain alpha and beta, myosin light chain 2 and 7, and transcriptional factor Nkx2.5) in EBs cultivated under non-adherent conditions for 5 days. The ratio of cardiomyocytes per other cells was also potentiated in EBs cultivated in non-adherent conditions for only 5 days followed by cultivation in adherent serum-free culture conditions. Nevertheless, the alteration in the percentage of beating foci among these two tested cultivation conditions vanished at later phases and also did not affect the total number of cardiomyocytes determined as myosin heavy chain positive cells at the end of the differentiation process on day 20. Thus, although these modifications of the conditions of ES cells differentiation may intensify cardiomyocyte differentiation, the final count of cardiomyocytes might not change. Thus, serum depletion was identified as a key factor that intensified cardiomyogenesis. Further, the treatment of EBs with N-acetylcysteine, a reactive oxygen species scavenger, did not affect the observed increase in cardiomyogenesis under serum depleted conditions. Interestingly, a mild induction of the ventricular-like phenotype of cardiomyocytes was observed in 5-day-old EBs compared to 8-day-old EBs. Overall, these findings bring crucial information on the mechanisms of ES cells differentiation into cardiomyocytes and on the establishment of efficient protocols for the cardiomyogenic differentiation of ES cells. Further, the importance of determining the absolute number of formed cardiomyocyte-like cells per seeded pluripotent cells in contrast to the simple quantification of the ratios of cells is highlighted.

## Introduction

The differentiation of pluripotent embryonic stem (ES) cells derived from the inner cell mass of blastocyst into various lineages represents an important tool to study the mechanisms underlying mammalian embryogenesis. ES cell cardiomyogenesis *in vitro* is a key technique in studies evaluating the molecular mechanisms of cardiomyogenesis and heart development, and also in the study of embryotoxicity [1–3]. In addition the differentiated cardiomyocytes obtained from ES cells may be used as an alternative source of neonatal cardiomyocytes in studies focused on the molecular background of heart diseases. Finally, cardiomyocytes derived from human ES cells are suggested as a potential source of transplantable cells for cell therapy [4, 5]. Due to extensive research, the development of cardiomyocytes from ES cells *in vitro* is a relatively well described and reproducible process [6–8]. However, a detailed understanding of cardiomyogenesis *in vitro* is still elusive and new approaches and methods to study all aspects of this process are required. Moreover, the precise direction of ES cell differentiation into cardiomyocytes, without employing genetic manipulation, in order to obtain populations of cells rich in highly differentiated cardiomyocytes is desirable for all the abovementioned applications.

Currently, the universal protocol employed for ES cell differentiation involves the formation of floating spheres—embryoid bodies (EBs) cultivated for several days under non adherent conditions, typically for seven to eight days. This is followed by the seeding of EBs onto adhesive tissue culture plastic and further cultivation. Typically, the beating foci of maturated cardiomyocytes appear within the adherent EBs layers after several days [6, 7]. However, cardiomyogenesis can also be observed in EBs cultivated the whole time in the form of floating EBs. The advantage of transferring EBs to adherent conditions allows easier observation of beating clusters or islands of maturated cardiomyocytes. However, the importance of the cultivation time for EBs in non-adherent and adherent conditions is unknown despite the fact that various factors clearly differ among these cultivation conditions, such as cell-to-cell contact, the microenvironment with the potential induction of hypoxia, and the local gradient of self-secreted growth factors that can all significantly influence the differentiation process.

Traditionally, all differentiation steps are performed in complete cell culture media containing fetal bovine serum (FBS) [6–8]. The major disadvantage of using FBS in differentiation cell culture media is the presence of unspecific FBS properties that have important effects on the progress of cardiomyogenesis *in vitro*. Thus, under extreme situations, some batches of serum may even inhibit ES cell cardiomyogenesis and cardiomyocyte maturation *in vitro* [9, 10]. Moreover, the presence of FBS may also induce the differentiation and expansion of cells of other undesirable non-cardiomyocyte lineages, including mesoderm and endoderm [11–13]. These unpredictable effects of FBS from different batches attributable to non-determinate FBS properties bring serious complications for the standardization of this procedure and the generalization of the obtained results. Therefore, alternative protocols for ES cell cardiomyocyte differentiation *in vitro* in serum-free conditions are being introduced and optimized [10, 14, 15]. Although most of these protocols still require the presence of FBS in the initial phase of EBs formation, the rest of the differentiation process is performed in media supplemented with growth factors and serum-replacements [16]. However, serum withdrawal or the absence of bone morphogenetic proteins (BMPs) during the initial phase of cardiomyogenesis, particularly during EBs formation, is currently suggested to be indispensable. Particularly important are the growth factors BMP-2 and BMP-4, which can mimic the effects of serum during the initial phase of cardiomyogenesis [15, 17–19]. Other notable growth factors have been described to improve cardiomyogenic processes in ES cells [20, 21].

Further, among other effects, FBS withdrawal can modulate the progress of ES cell cardiomyogenesis by the potential modulation of reactive oxygen species (ROS) production. According to Sauer, ROS are essential for the induction of cardiomyogenesis in ES cells [22, 23]. Interestingly, FBS removal during ES cell differentiation was found to increase ROS production [24–26]. Therefore, the modulation of ROS formation during ES cell differentiation can influence cardiomyogenic progress.

In the present work, we studied the modulation of mouse ES cells cardiomyogenesis *in vitro* by the modulation of FBS supplementation and the length of period of EB cultivation under non-adhesion conditions, and by the application of N-acetylcystein (NAC), a scavenger of ROS. Primarily, the occurrence of beating clusters and the expression of cardiomyocyte-specific genes (sarcomeric alpha actinin (Actn2), myosin heavy chain alpha and beta (Myh6 and 7), myosin light chain 2 and 7 (Myl2 and 7), and NK2 transcription factor related locus 5 (Nkx2.5) clearly show the potentiation of cardiomyogenesis by FBS depletion.

## Material and methods

### Cell culture and cardiomyocyte differentiation

Undifferentiated mouse ES R1 cells lines were adapted to feeder-free culture and routinely cultivated, as described previously [27]. R1_NKx2.5-GFP ES cells (clone NK4) carrying the Nkx2.5promotor-GFP reporter were prepared by the transfection of R1 cells with NKX2-5-Emerald GFP BAC reporter [28]. R1_ MHC-neor/pGK-hygro ES cells (clone HG8) carrying the Myh6 promotor regulating the expression of neomycin phosphotransferase were prepared by the transfection of R1 cells by MHC-neor/pGK-hygro plasmid (kindly donated by dr. Loren J. Field, Krannert Institute of Cardiology, Indianapolis, US [29])[30]. Both R1 subclones undergo cardiomyogenesis in the same manner as the maternal cell line, which was verified in our preliminary experiments (not shown).

Cells were cultivated in DMEM media supplemented with 15% FBS, 100 IU/ml penicillin, 0.1 mg/ml streptomycin, and 1x non-essential amino acid (all from Gibco-Invitrogen) and 0.05 mM β-mercaptoethanol (Sigma), referred to here as complete DMEM media, supplemented with 1 000 U/ml of leukemia inhibitory factor (LIF, Chemicon), referred to here as ES media. Cardiomyogenic differentiation was induced by the seeding of ES cells to form floating EBs. EBs formation was induced by ES cell cultivation on bacteriological dishes coated with 1% agar or by seeding ES cells in the form of hanging drops (400 cells per 0.03 ml drop) in complete ES media without LIF. This part of the differentiation process is referred to in this work as the inducing non-adherent phase of differentiation. In the next step, growing EBs were transferred onto adherent tissue culture dishes and cultivated in DMEM-F12 (1:1) supplemented with insulin, transferrin, selenium (ITS, Gibco-Invitrogene), and antibiotics (as above), i.e. the serum-free medium. This part of the differentiation process is referred to in this work as the differentiating adhesion phase. The cell culture medium was replaced by a fresh one every 2 days ADDIN. For beating analyses, EBs were seeded individually onto a 24-well plate. Beating was observed using an inverted tissue culture microscope and EBs were recognized as beating if spontaneous contraction was observed.

The effects of adhesion, FBS presence, and NAC treatment were tested as follows. Media with or without serum and with or without NAC were employed. After 8 days of differentiation overall, cells were lysed for mRNA isolation and the qRT-PCR analysis of cardiomyogenic markers. In detail, the differentiating conditions were as follows. A first group of day-5 EBs were seeded onto gelatinized tissue culture plastic in the presence or absence of serum and with or without 5mM NAC, and cultured for the next 3 days. A second group of ES cells were cultured as floating EBs for the whole period of 8 days; however, for the final 3 days, the medium was changed according to the same principle applied to the first group.

### Counting of cardiomyocytes

The relative number of cardiomyocytes in differentiating ES cell cultures was determined using the R1_NKx2.5-GFP cells mentioned above. Before analyses, cells were washed with phosphate buffered saline (PBS), incubated in a 0.3% solution of Collagenase II (Gibco) in DMEM media without serum for 20 minutes, and then incubated in trypsin (0.25% in PBS-EDTA, Gibco) for 5 minutes. Enzymes were inactivated by washing the cells with complete DMEM media and analysed on an Accuri flow-cytometer (BD Biosciences). The final ratio of cardiomyocytes to seeding cells was determined on the basis of the staining of cells according to the following. The EBs (each embryoid bodiewas generated from 400 cells) were treated as above and seeded onto gelatinized cell culture wells. After 2 days, cells were wash with PBS, fixed with 4% formaldehyde, permeabilized by 0.1% TWEEN 20 solution in PBS, stained using anti-cardiomyocyte heavy myosine antibody (anti-MHC, clone MF20, kindly provided by Dr. Donald Fischman, Developmental Studies Hybridoma Bank, Iowa City, IA, USA), and visualized using anti-mouse IgG conjugate Alexa488 (Invitrogen). Nuclei were counterstained with DAPI (1 mg/l). Images were acquired using an Olympus IX-51inverted fluorescence microscope (Olympus)[30].

The difference in cardiomyocyte mass was also determined using the HG8 cell line, see above. Cells were differentiated as mentioned above and on day 14 the Myh6-positive cardiomyocytes were selected by G418 antibiotic for six days [30]. ATP quantification in the whole cell lysate was employed to quantify the relative amount of viable selected cells [31].

### Analysis of gene expression by qRT-PCR

Total RNA was extracted by RNeasy Mini Kit (Mo-Bio). Complementary DNA was synthesized according to the manufacturer’s instructions for M-MLV reverse transcriptase kit (Sigma-Aldrich). qRT-PCR was performed in a Roche Light-cycler using the following program: an initial activation step at 95°C for 5 min, followed by 40 cycles at 95°C for 10 s, an annealing temperature (see below) for 10 s, and 72°C for 10 s. The gene expression of each sample was expressed in terms of the threshold cycle normalized to glyceraldehyde-3-phosphate dehydrogenase expression (*GAPDH*), as described previously [27] ADDIN. Primer sequences, annealing temperatures, and product lengths are listed in Table 1.The levels of Myl2 and Myl7 transcript were determined using a LightCycler Probes Master with UniversalProbe library probes (all from Roche, Germany) accordingto the manufacturer’s instructions. Ribosomal protein L13A (RPL13A) was used as a reference gene [32]; primer sequences and probes are listed in Table 2.

**Table 1:** Sequence of primers used in quantitative RT-PCR.

Gene of interest	Forward primer 5´→ 3´	Reverse primer 5´→ 3´	Annealing temperature (°C)	Product size (bps)
*Actn2*	ACCCAGCGCCATGAATCAGATAGA	TTCACTCCCTTGCTGGCTATGT	62	348
*Myh6*	GGGACTTCCGGCAGAGGTAT	ATCTCGCATCTCCTCGAGCA	61	188
*Myh7*	ACGTTTGAGAATCCAAGGCTCAG	ATCATCCAGGAAGCGTAGCG	62	366
*Nkx2*.*5*	AGGGGTGGGCAGCACCACT	CAGCGCGCACAGCTCTTTTT	66	405
*GAPDH*	AAGGGCTCATGACCACAGTC	CATACTTGGCAGGTTTCTCCA	62	252

**Table 2:** Probes and sequences of primers used in quantitative RT-PCR.

Gene of interest	Forward primer 5´→ 3´	Reverse primer 5´→ 3´	UPL probe no.
*RPL13A*	CATGAGGTCGGGTGGAAGTA	GCCTGTTTCCGTAACCTCAA	#25
*Myl2*	CCCAGATCCAGGAGTTCAAG	CTGCAGCCGCAGTAGGTT	#95
*Myl7*	CCCATCAACTTCACCGTCTT	AACATGCGGAAGGCACTC	#7

### Analysis of Ca^2+^ flow in beating cardiomyocytes

Cardiomyocytes were washed with Tyrode solution consisting of 140 mM NaCl, 5 mM KCl, 5 mM HEPES, 1 mM NaH_2_PO_4_, 1 mM MgCl_2_, 1.8 mM CaCl_2_ and 10 mM glucose (pH 7.4). Cells were loaded with 0.01 mM Fluo-4 AM dye (Life Technologies—Molecular Probes, F14201) in darkness at room temperature (RT) for 1h. Following dye loading, the cells were washed with Tyrode solution and further incubated for 20 min at RT. Imaging was performed on an Olympus IX-51 inverted fluorescence microscope with an Olympus SP-500UZ camera. The beating frequency and the width of individual peaks were analyzed from recordings by a CBAnalyser as described previously [30].

### Electrophysiological measurements

Recordings were made following Bébarová et al. [33]. Briefly, the whole cell patch-clamp technique in current clamp and voltage clamp modes was used (Axopatch 200B equipment, pCLAMP 9.2 software; *Molecular Devices*). The resistance of the filled glass electrodes was below 2 MΩ; the series resistance was compensated by up to 75%. The measured data were digitally sampled at 5 kHz. All recordings were performed at 37°C.

To elicit action potentials, suprathreshold current pulses (0.5 ms, 4–10 nA) were applied at astimulation frequency of 1 Hz. The whole cell membrane ionic current was measured using a 5-s ramp protocol between -110 and +40 mV. The composition of solutions (in mM) was as follows: 1. The external Tyrode solution: NaCl 135, KCl 5.4, MgCl_2_ 0.9, HEPES 10, NaH_2_PO_4_ 0.33, CaCl_2_ 0.9, glucose 10 (pH 7.4 adjusted with NaOH); 2. the patch electrode filling solution: L-aspartic acid 120, KCl 15, MgCl_2_ 1, K_2_ATP 5, EGTA 1, HEPES 5, GTP 0.1, Na_2_-phosphocreatine 3 (pH 7.25 adjusted with KOH).

### Statistical analysis

Data are expressed as mean ± SEM. Statistical analysis was assessed by paired T-test, by ANOVA, and by the Kruskal-Wallis test, post-hoc Bonferroni, or Dunns test.Values of P < 0.05 were considered to be statistically significant.

## Results

### The amount of the differentiation period that ES cells spend in the form of non-adherent EBs affects the progress of cardiomyogenesis

**Fig 1:**
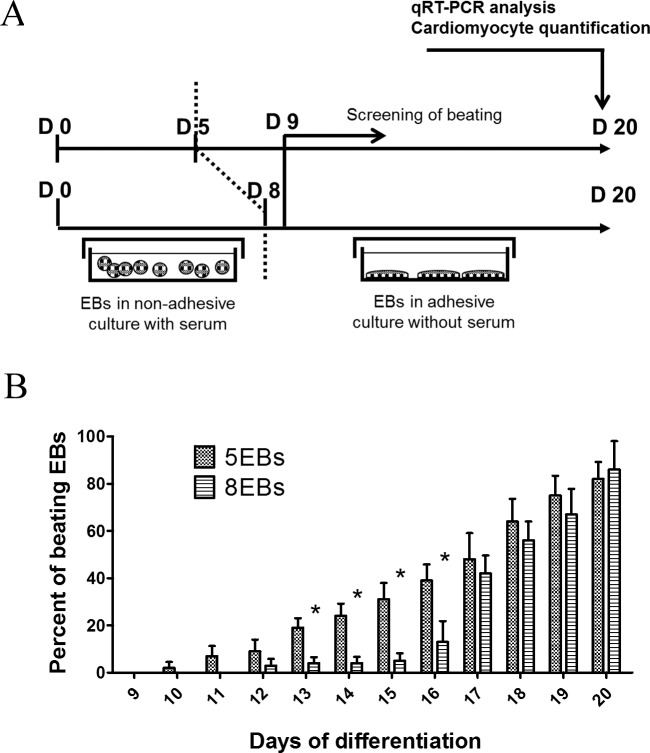
The experimental setup employed for comparison of the effects of different durations (5 vs. 8 days) of EBs cultivation under non-adherent conditions on cardiac differentiation analyzed by the determination of the percentage of beating EBs during cultivation and the expression of cardiomyogenic markers at the end of the differentiation experiment (A). Percentage of beating EBs in the adherent layer during the differentiation of mouse ES R1 cells that were primarily cultivated in the form of EBs under non-adherent conditions for 5 days or 8 days (B). Data are expressed as mean ± SEM (n = 4). Differences between samples were analyzed by ANOVA with Bonferroni's Multiple Comparison Test and considered statistically significant for p ≤ 0.05; they are marked with asterisks.

Primarily, the effect on the progress of cardiomyogenesis of the length of the cultivation period for differentiating mouse ES cells in the phase of non-adherent EBs in the presence of FBS was evaluated. The appearance of beating foci was analyzed in cells originating from EBs cultivated for 5 or 8 days before the seeding of cells into the serum-free adherent phase (5EBs and 8EBs protocol respectively, Fig 1A). In the case of cells originating from 5EBs, the first appearance of beating was observed on day 10 of differentiation, which means 5 days in the adherent phase (Fig 1B). Further, the number of beating EBs in the monolayer of cells originating from 5EBs increased linearly in time till the last day, day 20, of the experimental period. In contrast, the first appearance of beating in cardiomyocytes originating from 8-day-old EBs was observed on day 12 of differentiation, which means after 4 days in the adherent phase. Moreover, the appearance of beating was sporadic in cells from 8EBs until day 17 of differentiation, which means 9 days after re-seeding in adherent serum-free conditions. Interestingly, in this case, detectable beating foci were observed in this time period 12 days after seeding. Finally, the number of beating cells was comparable in differentiating cell cultures originating from 5EBs and 8EBs at the end of the experimental period, i.e. 20 days after the induction of differentiation (Fig 1B).

**Fig 2:**
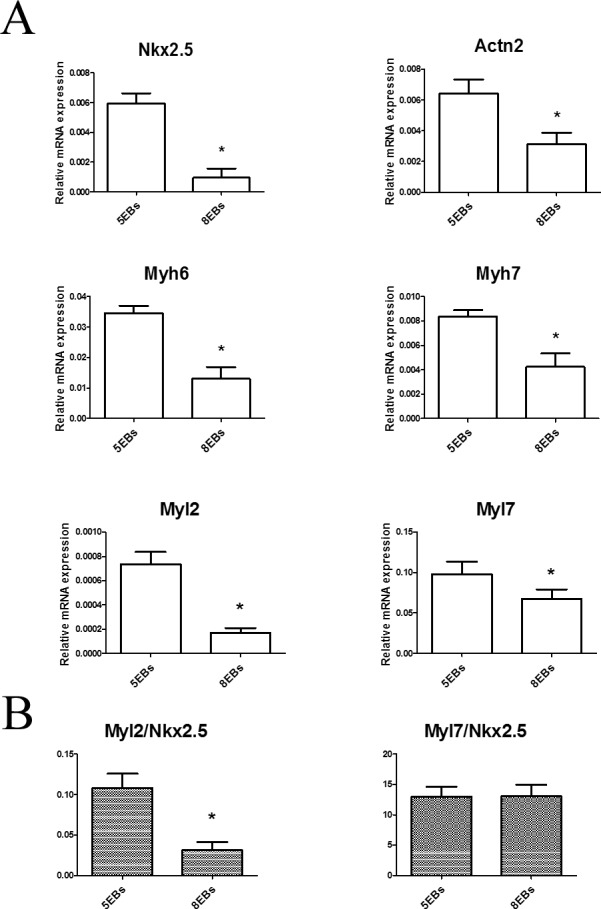
Gene expressions of transcriptional factor Nkx2.5, Actn2, Myh6, Myh7, Myl2 and Myl7, recognized as markers of cardiomyocyte differentiation in mouse ES R1 cells that were differentiated for 20 days *in vitro* and primarily cultivated in the form of EBs under non-adherent conditions for 5 days or 8 days (A). The ratios of Myls expression to Nkx2.5 expression are also shown (B). Data are expressed as mean ± SEM (minimum n = 4). Differences between samples were analyzed by paired T-test and considered statistically significant for p ≤ 0.05; they are marked with asterisks.

These data suggest a higher cardiac differentiation status in cells derived from 5EBs compared to cells from EBs cultivated for 8 days under non-adherent conditions. Thus, the relative level of cardiomyogenic differentiation status between cells originating from 5EBs and 8EBs was further analyzed on the basis of the expression of cardiomyocyte specific genes, transcription factor Nkx2.5, contractile apparatus protein sarcomeric α actinin (Actn2), myosin heavy chain alpha (Myh6) and beta (Myh7), and myosin light chain 2 (Myl2) and 7 (Myl7). Interestingly, confirming beating activity as a marker of cardiomyocytes, the mRNA levels of all these markers were significantly higher in differentiated cells prepared from EBs cultivated in non-adherent conditions for 5 days (5EBs) compared to their counterparts prepared from EBs cultivated under non-adherent conditions for 8 days EBs (8EBs). These mRNA levels were determined at the end of the experimental period after 20 days of overall differentiation (Fig 2A). To suggest the phenotype of developing cardiomyocytes, the ratios of Myl2 to Nkx2.5 expression levels and Myl7 to Nkx2.5 expression levels were calculated. The ratio in the case of Myl2 was higher in cells derived from 5EBs than in cells derived from 8EBs. The ratio in the case of Myl7 was the same for cells derived from both 5 and 8EBs (Fig 2B).

### The induction of accelerated cardiomyogenesis by shortening the non-adhesion phase did not yield an increase in the final number of differentiated cardiomyocytes

**Fig 3:**
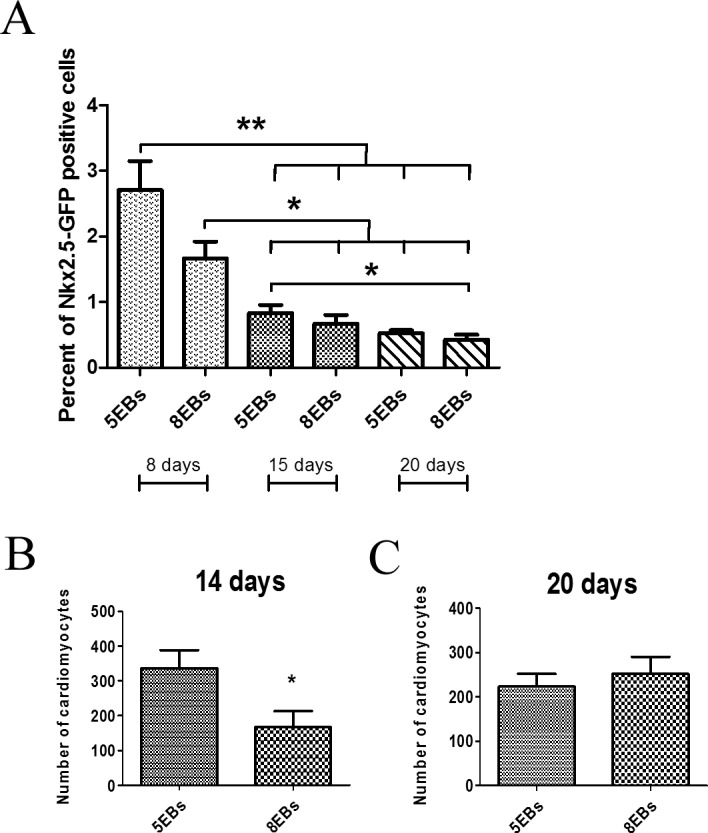
Flow-cytometric analysis of the Nkx2.5-GFP positive cell ratio in cultures derived from 5- and 8-day-old EBs. Samples were analyzed on days 8, 15 and 20 of differentiation (A). Number of cardiomyocytes formed per one EB (400 cells per EB were seeded) on days 15 and 20 of differentiation (B, C). Data are expressed as mean ± SEM (n = 4). Differences between samples were analyzed by ANOVA with Bonferroni's Multiple Comparison Test (A) and paired T-test (B, C) and considered statistically significant for p ≤ 0.05; they are marked with asterisks.

**Fig 4:**
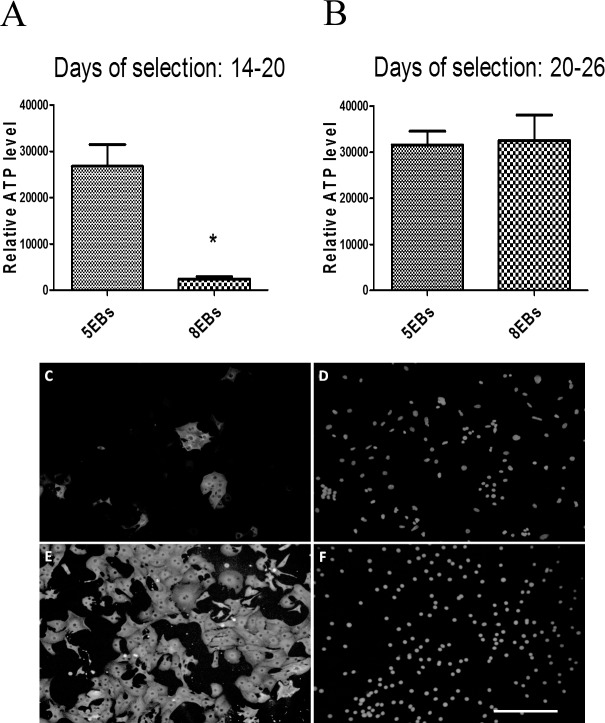
A number of viable cardiomyocytes determined as the level of ATP in the HG8 clone of the R1 ES cell (see methods) ratio in the culture derived from 5- and 8-day old EBs (A and B). Selection of cardiomyocytes by G418 antibiotics started on days 14 (Days of selection: 14–20) and 20 (Days of selection: 20–26) of differentiation, and continued for 6 days in both cases. Data are expressed as mean ± SEM (n = 4). Differences between samples were analyzed by paired T-test and considered statistically significant for p ≤ 0.05; they are marked with asterisks. The purity of the selected cardiomyocytes is also shown. Representative picture of non-selected (C, D) and selected (E, F) cardiomyocytes stained using the anti-MHC antibody MF20 (C, E) on day 20 of differentiation. Cell nuclei are counterstained by DAPI (D, F). Scale bar = 100 μm.

The number and percentage of forming cardiomyocytes was evaluated. According to cardio specific transcript expression (described above) and the flow-cytometric analysis of Nkx2.5-GFP positive cells (Fig 3A), the relative amounts of cardiomyocytes in 5EBs and 8EBs on days 8, 15 and 20 were determined. The proportional representation of cardiomyocytes decreased from 2.5% of Nkx2.5-GFP positive cells on day 8 to 1% on day 15 and to approximately 0.5% on day 20 (Fig 3A). Further, determination of the number of cardiomyocytes per number of seeded cells revealed a higher number of cardiomyocytes in cells derived from 5EBs compared to cells from 8EBs on day 15 of differentiation (350 cardiomyocytes per one EB formed from 400 ES cells in 5EBs, and approximately 200 cardiomyocytes per one EB in 8EBs). However, we did not observe any difference in cardiomyocyte number between the tested groups on day 20 of differentiation. In this case, the number of cardiomyocytes was determined approximately to be 200 cardiomyocytes per one EB in both cells derived from 5EBs and 8EBs (Fig 3B and 3C).These results were confirmed by experiment with selected cells expressing cardiomyocyte-specific Myh6. Interestingly, when cardiomyocyte selection was started on day 14 of differentiation, we obtained a significantly higher number of viable cells determined by ATP levels in cardiomyocytes derived from 5EBs (Fig 4A). In the case of selection starting on day 20 of differentiation, the number of viable cells was identical (Fig 4B).The effectivity of cardiomyocyte purification is also shown here (Fig 4C–4F).

### Physiological properties of formed cardiomyocytes

**Fig 5:**
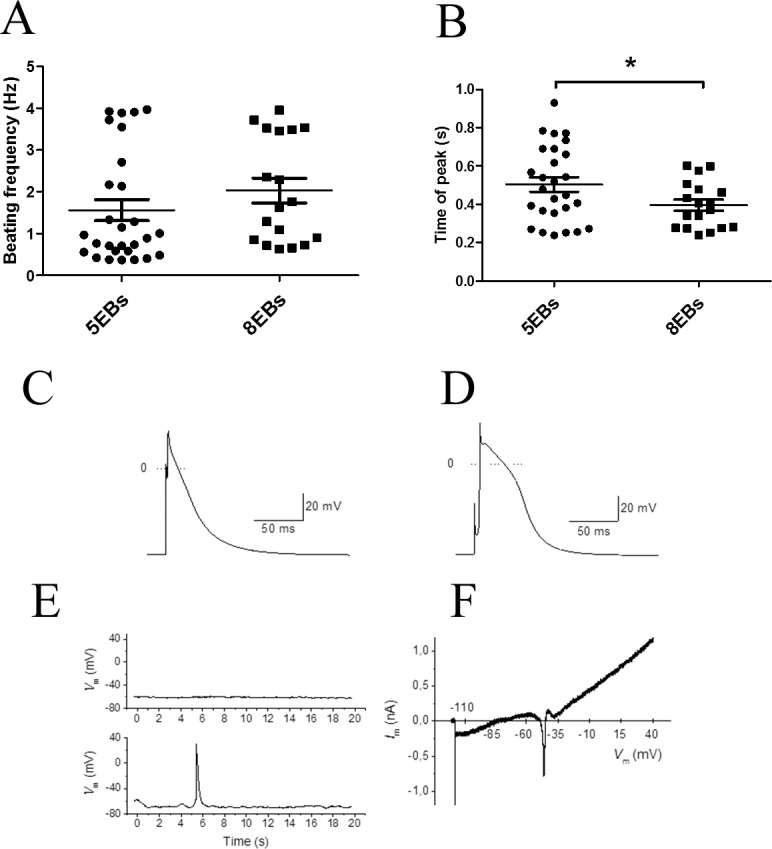
Electrophysiological characteristics of differentiated cardiomyocytes. Beating frequency (A) and duration of peak width (B) analyses by Ca^2+^ flux in formed 5EB and 8EB cardiomyocytes on day 20 of differentiation. Data are expressed as mean ± SEM (n = a minimum of 4). Differences between samples were analyzed by paired T-test and considered statistically significant for p ≤ 0.05; they are marked with asterisks. Representative action potential recordings from cells stimulated with suprathreshold current pulses (0.5 ms, 4–10 nA) at 1 Hz; a cells with atrial (C) and ventricular (D) action potential morphology. In the majority of cells, we did not observe any spontaneous electrical activity during 20-s recordings (E; upper panel); occasionally, a single spontaneous AP appeared during this period (E; lower panel); *V*_m_−membrane voltage. Whole cell membrane current during a 5-s ramp pulse at voltages between -110 and +40 mV (F); *I*_m_−membrane current.

To compare 5EBs and 8EBs cardiomyocytes, the Ca^2+^ flux kinetic was determined. The average value of the beating frequency was about 2 Hz and was similar for both 5EBs and 8EBs cardiomyocytes (Fig 5A). However, the duration of the contraction peak was significantly longer in 5EBs cardiomyocytes (an average value of 0.5 s in 5EBs versus 0.4 s in 8EBs). The distribution of cardiomyocytes according to the duration of the peak showed that fast beating cardiomyocytes (duration of peak ~ 0.2s) were present in both groups. However, slow beating cardiomyocytes (duration of peak > 0.6s) were found in 5EBs only (Fig 5B).

To further characterise the developing cardiomyocytes, electrophysiological measurements employing the whole cell patch-clamp technique in current clamp and voltage clamp modes was used. Most cardiomyocytes that were analysed exhibited the action potential morphology characteristic of atrial cardiomyocytes (Fig 5C). Rarely, a ventricular action potential morphology in 5EBs cardiomyocyte was also observed (Fig 5D). However, we were not able to measure the action potential using 20-day differentiating cardiomyocytes. For its determination, cardiomyocytes maturated for at least 30 days had to be used. However, only a limited number of such cardiomyocytes were able to maintain their membrane integrity and action potential within patch clamp measurements (Fig 5E and 5F).

### The role of particular culture conditions in the acceleration of cardiomyogenesis

#### The importance of adhesion for ES cell cardiomyogenesis

**Fig 6:**
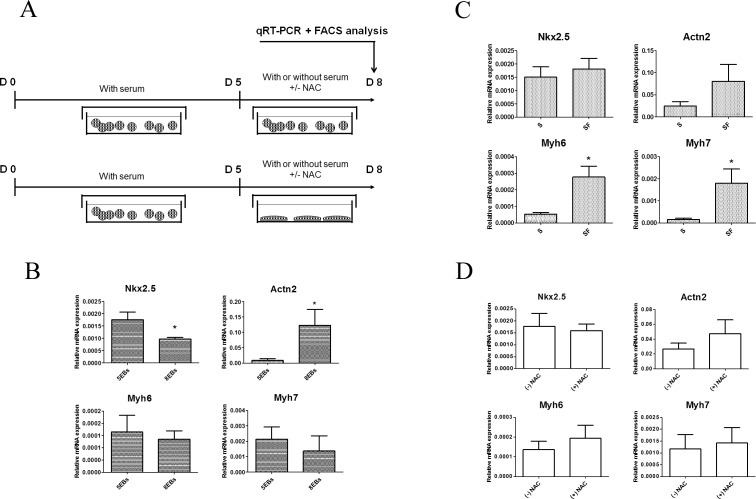
The experimental setup employed for comparison of the effects of adhesion, FBS supplementation, and NAC supplementation analyzed by determination of the expression of cardiomyogenic markers at the end of the differentiation experiment (8 days). Supplementation was altered for the last 3 days of cultivation under adherent or non-adherent conditions for EBs cultivation, after 5 days of cultivation in the form of EBs in the presence of FBS (A). Gene expressions of transcriptional factor Nkx2.5, Actn2, Myh6, and Myh7, recognized as markers of cardiomyocyte differentiation–data are presented as the effect of individual variables: the adhesion of EBs on days 5 or 8 of differentiation (B), the presence (S) or absence (SF) of FBS (C), and the presence ((+)NAC) or absence ((-)NAC) of N-Acetyl Cysteine (D). Data are expressed as mean ± SEM (n = 4). Differences between samples were analyzed by Kruskal-Wallis test and post-hoc Dunns test and considered statistically significant for p ≤ 0.05; they are marked with asterisks.

To further clarify the importance of adhesion for the above observed phenomenon, cardiomyogenic differentiation in ES cells cultivated in the form of floating EBs for the whole differentiation period was compared with that for cells cultivated in the form of floating EBs for 5 days with the subsequent transfer of EBs to adherent culture conditions. Cardiomyogenic differentiation was only observed for 8 days overall (see Fig 6A for a diagram of the experimental setup). Interestingly, the cultivation of EBs in non-adherent conditions for the whole 8-day period suppressed Nkx2.5 expression and supported Actn2 expression. Differences in the levels of Myh6 and Myh7 transcripts were insignificant between the experimental groups (Fig 6B). This suggests that adhesion alone increased cardiomyogenesis.

#### The presence of FBS during the EBs adhesion phase of differentiation inhibits cardiomyogenesis

Next, the importance of serum withdrawal was tested. ES cells were cultivated under non-adherent conditions for 5 days in the presence of 15% FBS. Then, EBs were transferred to adherent conditions and cultivated in the absence and presence of 15% serum; subsequently, cardiomyogenic differentiation was only observed for the next 3 days (i.e. 8 days overall; see Fig 6A for a diagram of the experimental setup). Interestingly, the serum-free conditions significantly potentiated the expression of both Myh6 and Myh7 (Fig 6C). The expression of the other cardio-specific markers Nkx2.5 and Actn2 level were unaffected.

#### NAC did not affect cardiomyogenesis in our model

The effect of NAC, a compound with the potential to eliminate intracellular increases in ROS formation, was also tested. NAC supplementation was applied for the last 3 days of cell culture under FBS free conditions, i.e. for the last 3 days of the 8-day period of differentiation (see Fig 6A for a diagram of the experimental setup). However, NAC was not revealed to have any effect on the expression of any determined marker of cardiomyogenic differentiation (Fig 6D).

**Fig 7:**
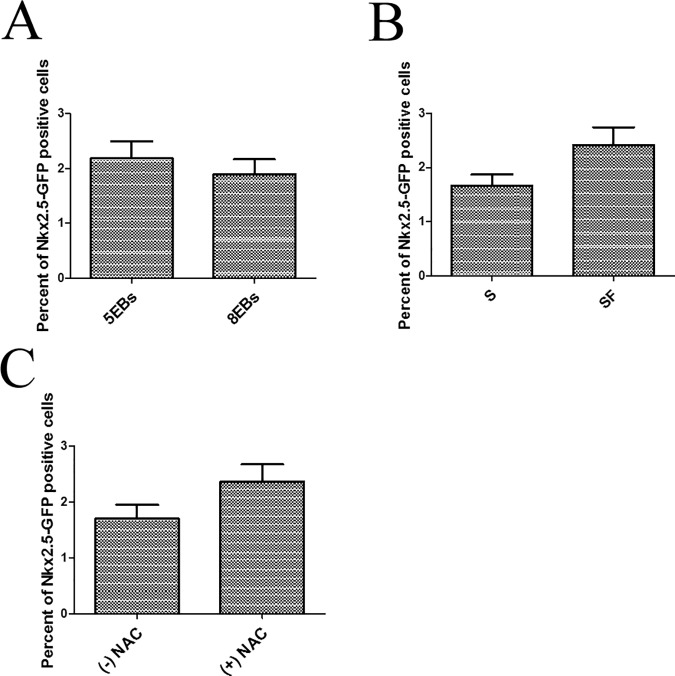
Flow-cytometric analysis of the Nkx2.5-GFP positive cell ratio in cells cultured as described in Fig 6A. Samples were analyzed at the end of the differentiation experiment (8 days). Data are presented as the effect of individual variables: the adhesion of EBs on days 5 or 8 of differentiation (A), the presence (S) or absence (SF) of FBS (B), and the presence ((+)NAC) or absence ((-)NAC) of N-Acetyl Cysteine (C). Data are expressed as mean ± SEM (n = 4). Differences between samples were analyzed by Kruskal-Wallis test and post-hoc Dunns test and considered statistically significant for p ≤ 0.05.

The percentage of forming cardiomyocytes was also evaluated. The results of the flow-cytometric analysis of Nkx2.5-GFP positive cells in 5EBs and 8EBs cultured as described in Fig 6A, which was performed on day 8 of differentiation, were evaluated and found to be in accord with the cardio-specific transcript expression (described above). We observed no significant effect of adhesion (Fig 7A), serum (Fig 7B), or NAC (Fig 7C) on the amount of Nkx2.5-GFP positive cells found here.

## Discussion

In this study, the cardiomyocyte maturation of mouse ES cells was accelerated to a period of only 5 days by a reduction in the cultivation of EBs under non-adherent conditions and in the presence of FBS, followed by cultivation under adherent conditions in serum-free media. Moreover, we also observed the slightly improved formation of ventricular-like cardiomyocytes through such shortening of the non-adherent phase of ES cell differentiation. Interestingly, the acceleration of cardiomygenesis did not affect the final overall number of cells expressing cardiomygenic markers; instead, it had an influence on the progress and final stage of cardiomygenesis. Further, the expression of Actn2 was affected differently compared to the expressions of Myh6 and Myh7 by alterations in the presence of serum and the conditions for adherence. This suggests different regulation of the expressions of distinct components of the contractile apparatus during cardiomyogenesis. These phenomena require further study.

As summarized previously, the basic protocol for ES cell differentiation involving EBs cultivation under non-adherent conditions combined with the transfer of EBs into adherent conditions typically suggest seven to eight days of cultivation under the primary phase in non-adherent conditions. Preliminary modifications of this protocol showed that in cells derived from EBs transferred to adherent conditions after just 4 days cardiomyogenic differentiation remains, as documented by the appearance of beating foci (data not shown). However, a high level of heterogeneity under these conditions was observed, which can probably be related only to limited development, which could be connected with the rapid spreading of cells after transfer to adherent conditions. Thus, for this study, the cultivation of EBs under non-adherent conditions for 5 days was selected as the earliest tested period. Interestingly, as presented herein, the overall frequency of beating EBs (beating foci) was similar for EBs cultivated under non-adherent conditions for 7–8 days [17, 34]. However, although the final frequency of the appearance of beating foci was the same for 5- and 8-day EBs there was a significant positive shift in the progress of the appearance of beating foci in cells derived from EBs cultivated under non-adherent conditions for 5 days. Interestingly, this was accompanied by the significantly higher expression of cardiomyogenic specific genes at the end of cultivation (20th day) in the case of 5-day EBs cultivated under non-adherent conditions. The functional maturity of cardiomyocytes was also tested by determining simple beating characteristic in cardiomyocytes derived from 5EBs and 8EBs. The beating frequency and beat peak width which corresponded to the speed of an individual pulse from digital video records of Ca^2+^ flux were determined. The beating frequency was the same in both groups of cardiomyocytes. However, the duration of the contraction peak was significantly longer in some 5EBs cardiomyocytes compared to 8EBs cardiomyocytes. This is in accord with the higher expression of most ventricular Myl2 [35–37] in 5EBs cardiomyocytes, which mark the appearance of ventricular-like cardiomyocytes in this group.

Various factors have been determined to affect the process of cardiomyogenesis in differentiating mouse ES cells. Among them are the level of ROS formation, mechanical strain [38, 39], or the presence of growth factors [10, 15, 40]. The withdrawal of growth factors and hormones contained in FBS not only omits the presence of growth factors but also induces the formation of intracellular ROS formation [25, 26]. Further, cell adhesion can mediate a mechanical strain [41, 42]. The withdrawal of growth factors contained in FBS is the key factor influencing ES cell differentiation [11, 12]. Data suggest that serum depletion leads to the early appearance of beating foci and the acceleration of Myhs and Myls expression, which we considered as increasing differentiation. Based on these facts, the depletion of serum seems to be key factor improving the differentiation and maturation of cardiomyocyte progenitors. As mentioned above, it can be expected that serum-free conditions may both repress the expansion of other mesodermal or endodermal cells in culture [11, 12, 40] and may also directly affect cardiomyogenesis [10, 14]. Further, our extended experiments showed that the transfer of EBs to adherent conditions increased Nkx2.5 expression in particular, which can be related to the increased presence of cardiomyocyte progenitors or the maturation status of these cells. We observed no difference in the proportions of Nkx2.5-GFP positive cells in these groups (Fig 7A). Thus, we suggest that adhesion can improve the maturation of precursors of cardiomyocyte progenitors in EBs. In contrast, the withdrawal of FBS increased the expression of both Myh6 and Myh7. Interestingly, in contrast to expectation, the application of NAC as a ROS scavenger was without effect. Thus, the importance of increased ROS production for the cardiomyogenesis of ES in the employed model of EBs could not be confirmed for this time window of differentiation.

Interestingly, the potentiated cardiomyogenesis in cells derived from 5EBs was documented by the higher number of cardiomyocytes in the early phase of differentiation, which correlated with the higher number of beating EBs compared to cells derived from 8EBs. However, the same number of cardiomyocytes was determined on the last day (day 20) of the experiment in both groups (5EBs versus 8EBs). Importantly, the direct counting of cardiomyocytes can be connected with several uncertainties. Cardiomyocytes form compact clusters similar to the functional syncitium of myocard; quantitative isolation is thus complicated by the potential loss of cells or whole clusters. To verify our results, the indirect quantification of cardiomyocytes selected on the basis of their gene expression was performed[30]. The increased level of cardiomyocyte markers in mixed differentiation populations is extrapolated from the number of other generated cell types within prolonged EBs differentiation. We also can suggest that the major part of cardiomyocyte progenitors are formed within the first 5 days of ES cell differentiation.

Remarkably, the data presented in the above paragraph document variability in the regulation of gene expressions of components of the cardiomyocyte contractile apparatus, particularly the expression of Actn2 compared to the expression of Myh6 and Myh7. As mentioned above, non-adherent conditions of EBs cultivation only promoted the expression of Actn2, without the induction of Nkx2.5 or Myh6 and Myh7 (Fig 6B). In contrast, serum withdrawal promoted the gene expression of Myh6 and Myh7 without a significant effect on Actn2 (Fig 6C). This points to the heterogeneity in signals regulating the particular steps of cardiomyocyte differentiation and maturation. Actn2 expression is widely employed as a marker of differentiation; however, the regulation of the expression of this gene has not been studied. Thus, according to current literature, there is no currently known mechanism to explain this observed difference.

In conclusion, we demonstrate here that simple manipulation with the length of EBs culture and the presence of serum can accelerate cardiomyogenesis in pluripotent ES cells. However, the final yield with respect to the number of cardiomyocytes did not change, and the observed effect reflects the dynamics of other non-specified cell types in culture. The results presented here may improve our view of the mechanism of *in vitro* cardiomyogenesis from pluripotent stem cells and suggest a possible way of manipulating such a process.
